# Monocyte Subpopulations from Pre-Eclamptic Patients Are Abnormally Skewed and Exhibit Exaggerated Responses to Toll-Like Receptor Ligands

**DOI:** 10.1371/journal.pone.0042217

**Published:** 2012-07-27

**Authors:** Ebtisam Al-ofi, Seth B. Coffelt, Dilly O. Anumba

**Affiliations:** 1 Academic Units of Reproductive & Developmental Medicine, University of Sheffield Medical School, Sheffield, United Kingdom; 2 Inflammation & Tumour Targeting, University of Sheffield Medical School, Sheffield, United Kingdom; VU University Medical Center, The Netherlands

## Abstract

The leading cause of pregnancy-associated mortality and morbidity is pre-eclampsia (PE). Although information regarding the etiology of this disease is scant, its pathophysiology is characterized by abnormal placentation, endothelial dysfunction as well as an exaggerated inflammatory response. Clinical evidence also indicates that the abundance of many immune cells at the feto-maternal interface and in the circulation of PE patients is abnormal, when compared with normal pregnant (NP) controls. In addition, the phenotype and function of some of these cells is altered. To further characterize the systemic effects of PE on circulating cells, we analyzed monocytic subpopulations in NP and PE patients by flow cytometry. We found that non-classical CD14^low^CD16^+^ monocytes are significantly increased in women with PE and they display irregular expression of several chemokine receptors and antigen presentation molecules. The most striking phenotypic difference among the cell surface molecules was the marked upregulation of TLR4 expression, where both CD14^high^CD16^+^ and CD14^low^CD16^+^ monocytes demonstrated higher levels than their NP counterparts. Stimulation of PE monocytes with TLR ligands resulted in profound secretion of various cytokines in comparison with NP controls. These data suggest that PE monocytes are hyper-responsive to TLR ligands and this may contribute to exacerbation of the disease.

## Introduction

Pre-eclampsia (PE) is a potentially life-threatening, pregnancy disorder characterized by new onset of hypertension and proteinuria after 20 weeks gestation [1]. The incidence of PE is common varying between 2–7% of healthy primigravid women [2]. Abnormal placentation [3], [4], endothelial dysfunction [5]–[7] as well as an exaggerated inflammatory response [8], [9] are thought to be the basis of the clinical manifestations of PE. However, the underlying cause(s) of PE remain unknown and its pathogenesis remains poorly understood.

During pregnancy, innate immune cells are abundantly present at the feto-maternal interface where they are thought to facilitate implantation, aid in placental development and maintain fetal tolerance [10]. The perturbation of this feto-maternal immune system has been recognized for some time now in PE patients. In the circulation, both monocytes and granulocytes display aberrant expression levels of surface antigens, cytokines and reactive oxygen species [11]–[14]. For example, monocytes display increased expression of CD14 and CD11b and produce more interleukin (IL)-1β, IL-6 and IL-8 than healthy pregnancy controls [11]–[14]. These phenotypic changes are thought to affect monocytes in several ways, including their adherence to the vasculature as well as a down-regulation in their immunosuppressive functions once localized at the feto-maternal interface [15]. In support of these notions, several studies have reported abnormal numbers of decidual macrophages [16]–[18] and decreased expression of the immunosuppressive cytokine, IL-10, in PE maternal blood serum compared to normal pregnancy controls [19], [20]. As monocytes and macrophages are critical for successful pregnancy, we set out to further characterize the monocyte phenotype in PE patients.

Monocytes in human peripheral blood can be divided into three distinct populations based on expression of the lipopolysaccharide (LPS) receptor, CD14, and the Fcγ-III receptor, CD16. The nomenclature and classification of these monocyte subsets has recently been updated into three populations: classical monocytes (CD14^high^CD16^−^), intermediate monocytes (CD14^high^CD16^+^), and non-classical monocytes (CD14^low^CD16^+^) [21]. Emerging evidence indicates that each subpopulation has a unique gene signature and may also have differential functions in inflammation and immunity [22], [23]; although, the hierarchical relationship and differentiation lineage between the subpopulations is still unclear.

In this study, we report that the proportion of non-classical CD14^low^CD16^+^ monocytes in PE patients is increased, while classical CD14^high^CD16^−^ monocytes are decreased in comparison with normal pregnant (NP) controls. The phenotype of PE monocytes was also altered, exhibiting aberrant expression of chemokine receptors, antigen presentation molecules, and Toll-like receptors (TLR). Moreover, treatment of PE monocytes with TLR ligands resulted in an exaggerated production of pro- and anti-inflammatory cytokines. These results provide further evidence that the phenotype of monocytes in PE patients is abnormal and this may exacerbate the problems associated with PE.

## Materials and Methods

### Ethics Statement

Ethical approval for this study was received from South Yorkshire Research Ethics Committee (09/H1310/12).

### Subjects

Women with established pre-eclampsia were diagnosed by the criteria of the International Society for the Study of Hypertension in Pregnancy (ISSHP) [24], and were recruited from the antenatal clinics and obstetric day care unit of the Jessop Wing, University of Sheffield. Healthy non-pregnant (Non-P) female volunteers were also studied to determine baseline non-gestational levels. The Non-P women had normal menstrual cycles and were not on hormonal contraception (**Table 1**). Our NP cohort included 10 Caucasian women and 1 black woman and the PE cohort included 15 Caucasian women and 2 black women. No study participants had any systemic infection or urinary tract infection. All participants gave informed written consent for 12 mL of fresh venous blood to be collected into a tube containing EDTA anticoagulant.

**Table 1:** Patient characteristics.

	Non-pregnant (n = 11)	Normal pregnant (n = 11)	Pre-eclampsia (n = 17)	*P* value
Age (years)	31.0±5.4	29.3±4.9	31.4±5.6	0.26
Gestational age (weeks)		32.0±3.7	32.9±3.7	0.38
Gravidity		1–4	1–4	
SBP (mm Hg)	110.0±10.0	114.0±11.0	150.4±8.6	0.0001
DBP (mm Hg)	70.0±10.0	70.0±10.0	98.3±4.2	0.0001
24 hour urine collection (g/24 hrs)	0	0	0.84±1.3	
Urine Dipstick Protein Test	0	0	(1+–3+)	

### Flow Cytometry

Peripheral blood mononuclear cells (PBMCs) were isolated from fresh venous blood layered over Ficoll-Pague Plus (Amersham Biosciences) which was centrifuged for 20 min at 800 × g. The white layer was removed and washed twice with HBSS +0.5% BSA as previously described [25]. The cells were blocked with 5 µL of anti-human Fc receptor blocking reagent (Miltenyi Biotic, Surrey) for 10 minutes at 4°C. PBMCs were labelled with the following monoclonal antibodies for 30 minutes at 4°C: anti-CD14-AlexaFluor700 (clone HCD14; Biolegend), anti-CD16-eFlour450 (clone CB16; eBioscience), anti-CCR2-PE (clone FAB151P; R&D Systems), anti-CCR5-FITC (clone HEK/1/85a; Biolegend), anti-HLA-DR-PerCP-Cy5.5 (clone LN3; eBioscience), anti-TIE2-APC (clone FAB3131A; R&D Systems), anti-TLR2-FITC (clone TL2.1; eBioscience), and anti-TLR4-PE (clone HTA125; eBioscience). PBMCs were also labelled with isotype negative controls. Cells were washed twice by cold BD flow cytometry stain buffer, before acquisition was done on a LSRII Flow Cytometer (BD Bioscience). DIVA software (BD Bioscience) was used for analysis. Compensation was calculated using anti-mouse compensation bead sets (BD Bioscience). A gate specific for the monocyte population was created on the forward scatter (FSC) and side scatter (SSC) dot plot and a total of 10,000 events were acquired. Monocyte sub-populations were identified according to CD14 and CD16 surface markers as defined previously [21], [26], [27].

### Monocyte Stimulation

Isolated PBMCs were seeded in 12-well tissue culture flat bottom plates at a density of 1.8 × 10^6^ cells/well. The cells were incubated with medium (IMDM supplemented with 2% FBS, 2 mM L-Glutamine and penicillin-streptomycin) at 37°C with 5% CO_2_ After two hours of incubation the medium was removed and the adherent monocytes were washed twice with cold PBS. The medium was then replaced. Cells were treated with 100 ng/ml Lipopolysaccharides (rough strains) from *Escherichia coli* (Sigma-Aldrich), 5 µg/ml Peptidoglycan from *Staphylococcus aureus* (Sigma-Aldrich) or left untreated for 24 hours. Conditioned medium was collected after this time and frozen at −20°C.

### Cytometric Bead Array

Cytometric bead array technique was used to measure IL-6, TNFα and IL-10 cytokines within monocyte conditioned medium. This assay was performed following the manufacturer’s instructions (BD Biosciences) at the Flow Cytometry Core Facility in the Sheffield University Medical School. BD FACS Array flow cytometry machine and FCAP Array software were used for analysis. To calculate fold change, the median fluorescence intensity (MFI) of LPS- or PDG-treated was divided over the unstimulated MFI.

### Statistical Analysis

Results are illustrated as mean ± standard error of the mean (SEM). Mann-Whitney *U*-test was used to compare differences between Non-P and NP or NP and PE using GraphPad Prism software version 5.0. *P* values < 0.05 were considered significantly different.

## Results

### Monocyte Subpopulation Frequency is Skewed in Pregnant Women with PE

**Figure 1:**
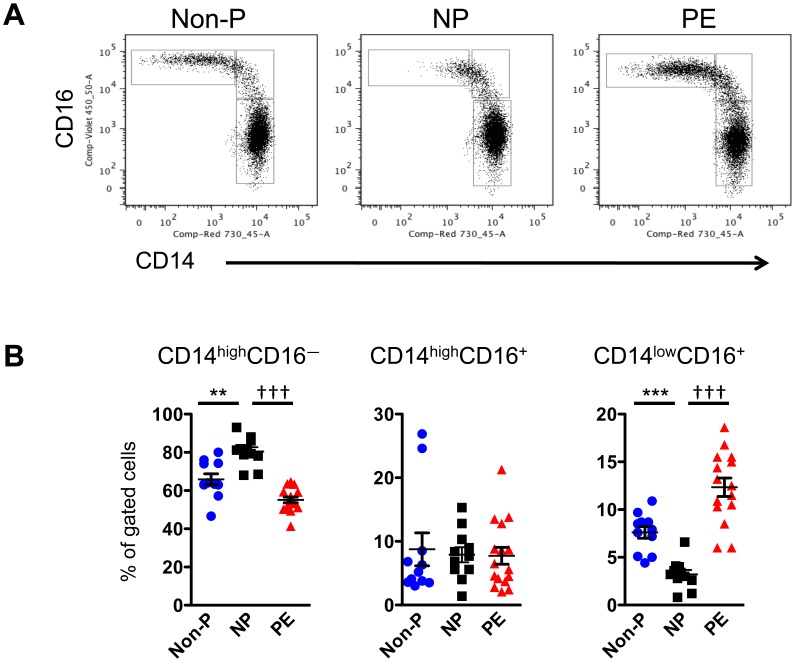
The proportion of monocyte subpopulations is skewed in patients with PE. PBMCs from non-pregnant (Non-P), normal pregnant (NP), and pre-eclampsia (PE) patients were stained with anti-CD14-AlexaFluor700 and anti-CD16-eFluor450 antibodies then analyzed by flow cytometry. (**A**) Dot plots showing CD14 and CD16 expression on gated monocytes. (**B**) Graphic representation of percentage of gated cells (mean ± SEM). Statistical significance was determined by Mann-Whitney *U* test (n = 11−17). **p < 0.01, ***p < 0.001 as compared with Non-P; ^†††^ *p* < 0.001 as compared with NP.

Blood samples were collected from non-pregnant (Non-P), normal pregnant (NP), and pregnant women with pre-eclampsia (PE). Peripheral blood mononuclear cells (PBMCs) were isolated from these samples, stained with anti-CD14 and anti-CD16 antibodies, and analyzed by flow cytometry. Gates were first placed around monocytes on FSC/SSC plots. No difference in the total number of monocytes amongst all PBMCs was noted between the three groups (data not shown). By contrast, differences in monocyte subpopulations were prevalent between the three groups (**Figure 1**). NP patients exhibited a greater proportion of CD14^high^CD16^–^ monocytes and a lower proportion of CD14^low^CD16^+^ monocytes when compared with Non-P patients. Interestingly, this pattern was reversed in women with PE, where the non-classical CD14^low^CD16^+^ monocytes were significantly increased over NP controls. No differences were observed in the intermediate CD14^high^CD16^+^ monocyte population between any of the patients.

### PE is Associated with Changes in Monocyte Phenotype

**Figure 2:**
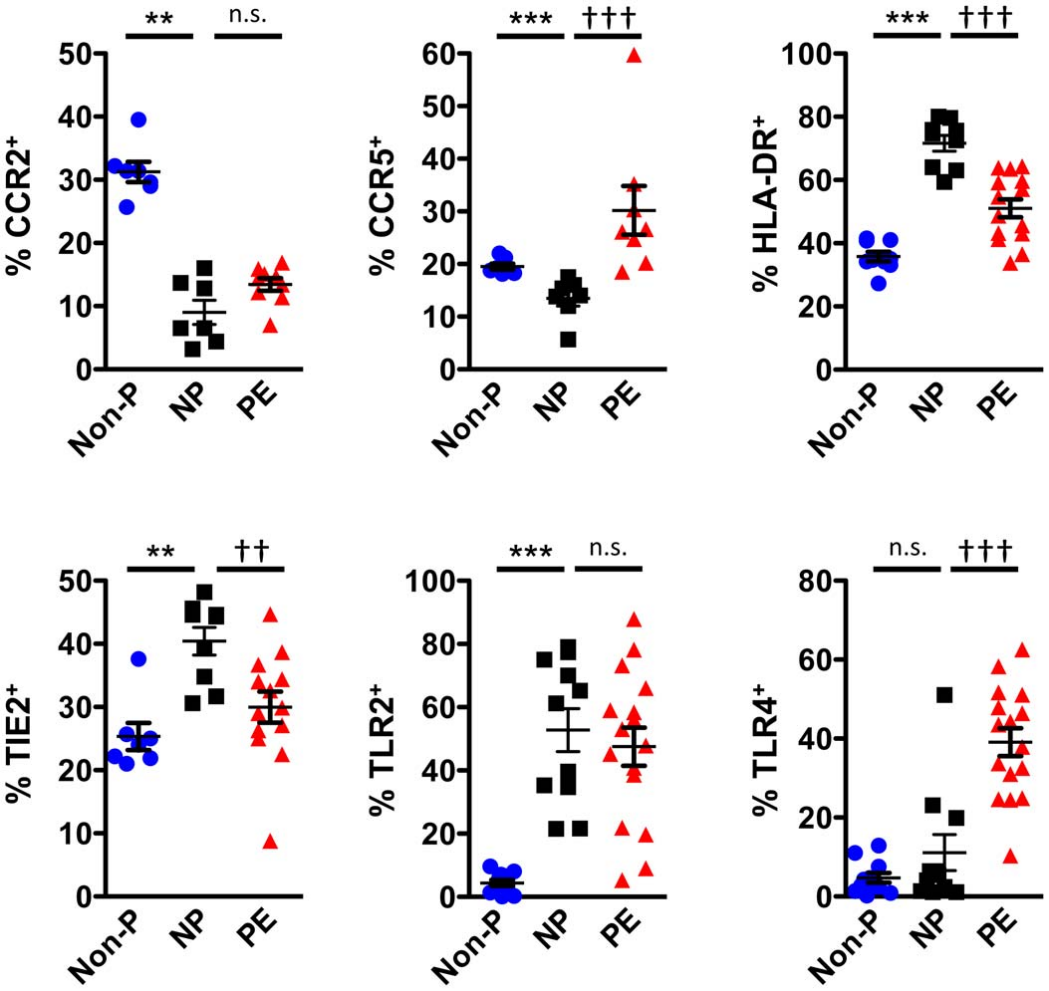
NP and PE monocytes display marked phenotypic differences. PBMCs from non-pregnant (Non-P), normal pregnant (NP), and pre-eclampsia (PE) patients were stained with anti-CD14-AlexaFluor700, anti-CD16-eFluor450, anti-CCR2-PE, anti-CCR5-FITC, anti-HLA-DR-PerCP-Cy5.5, anti-TIE2-APC, anti-TLR2-FITC, and anti-TLR4-PE antibodies then analyzed by flow cytometry. Gates were placed around monocytes on FSC/SSC plots and the percentage of positive cells was calculated based off isotype controls. Values are illustrated as mean ± SEM. Statistical significance was determined by Mann-Whitney *U* test (n = 5−16). n.s. =  not significant, **p<0.01, ***p<0.001 as compared with Non-P; ^†^ *p*<0.05, ^††^ *p*<0.01, ^†††^ *p*<0.001 as compared with NP.

Next, we investigated the phenotype of monocytes in Non-P, NP, and PE women. In relation to Non-P patients, the monocytes of NP patients expressed lower levels of CCR2 and CCR5, but higher levels of HLA-DR, TIE2 and TLR2 (**Figure 2**). The number of TLR4^+^ monocytes was equivalent between the two groups. When comparing NP and PE monocytes, PE patients displayed a reduced number of HLA-DR^+^ and TIE2^+^ monoyctes, whereas CCR5^+^ and TLR4^+^ monocytes were dramatically increased (**Figure 2**). A similar expression profile for CCR2 and TLR2 was observed between the two groups. These data indicate that monocytes from PE patients are phenotypically different from NP patients.

**Figure 3:**
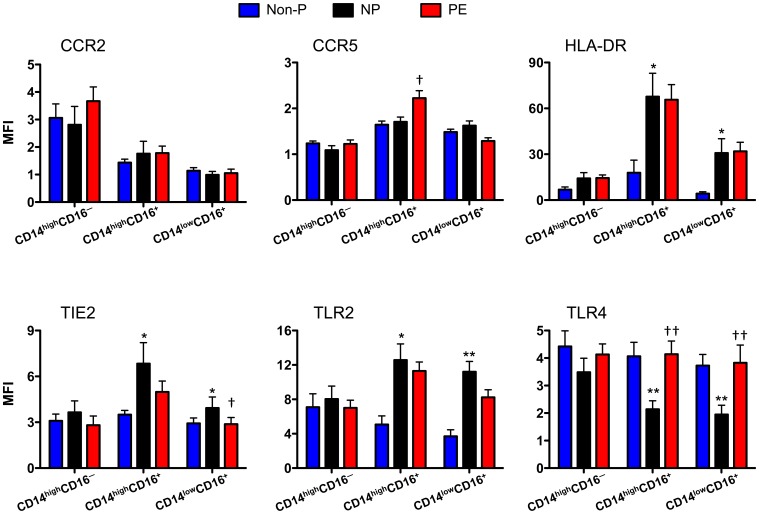
Expression of phenotypic markers on monocyte subpopulations. PBMCs from non-pregnant (Non-P), normal pregnant (NP), and pre-eclampsia (PE) patients were stained with anti-CD14-AlexaFluor700, anti-CD16-eFluor450, anti-CCR2-PE, anti-CCR5-FITC, anti-HLA-DR-PerCP-Cy5.5, anti-TIE2-APC, anti-TLR2-FITC, and anti-TLR4-PE antibodies then analyzed by flow cytometry. Gates were placed around CD14/CD16 monocyte subpopulations and median fluorescence intensity (MFI) was calculated based off isotype controls. Values are illustrated as mean ± SEM. Statistical significance was determine by Mann-Whitney *U* test (n = 7−17). *p<0.05, **p<0.01 as compared with Non-P; ^†^ *p*<0.05, ^††^ *p*<0.01 as compared with NP.

To determine whether the differences we observed were due to changes in expression by specific monocyte subpopulations, we examined the phenotypic markers on classical, intermediate, and non-classical subpopulations. Interestingly, classical monocytes did not exhibit any differences between the three patient cohorts. All the observed changes could be attributed to intermediate or non-classical monocytes. Expression levels of CCR2, HLA-DR, and TLR2 were not different among monocytic subpopulations of NP and PE patients (**Figure 3**). However, the intermediate CD14^high^CD16^+^ monocyte population of PE patients displayed higher expression of CCR5 and TLR4 than NP patients. TLR4 was also significantly increased on non-classical CD14^low^CD16^+^ monocytes from PE when compared with NP controls. Moreover, TIE2 expression was significantly reduced on the CD14^low^CD16^+^ monocytes subset in PE. These data suggest that phenotypic differences between NP and PE patients can be attributed to specific monocyte subpopulations.

### Monocytes from PE Patients Exhibit an Amplified Response to TLR Ligands

**Figure 4:**
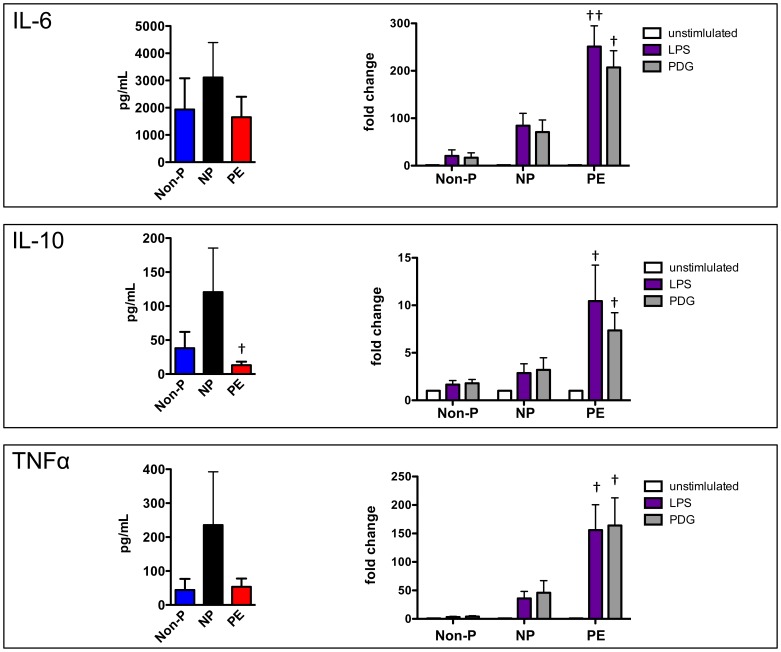
Monocytes from PE patients exhibit an amplified response to TLR ligands. Monocytes from non-pregnant (Non-P), normal pregnant (NP), and pre-eclampsia (PE) patients were isolated, seeded onto tissue culture plates, then treated with 100 ng/mL LPS or 5 µg/mL peptidoglycan (PDG) for 24 hours. Conditioned medium was collected after this time and analyzed by cytometric bead array to measure cytokine levels. ***left panels:*** Basal levels of cytokine production by monocytes. ***right panels*** *:* Graphic representation of the response of monocytes to LPS and PDG. Fold change was calculated by dividing MFI values over untreated control MFI values and illustrated as mean ± SEM. Comparisons were made between NP and PE fold changes. Statistical significance was determined by Mann-Whitney *U* test (n≥5). ^†^ *p*<0.05, ^††^ *p*<0.01 as compared with NP.

Since TLR4 expression was increased on PE monocytes, we asked whether the observed phenotypic changes have any consequence on monocyte activity. We treated monocytes from Non-P, NP, and PE patients with TLR4 ligands and monitored cytokine expression. Without stimulation, IL-6, IL-10 and TNFα release by PE monocytes was reduced in relation to NP; although, only IL-10 secretion was significantly reduced (**Figure 4**). Lipopolysaccharide (LPS) and peptidoglycan (PDG) significantly induced all three cytokines in each patient cohort when compared to unstimulated, control groups. However, TLR ligand-treated PE monocytes exhibited a marked and significant increase over that seen in TLR ligand-treated NP monocytes. Taken together, these data indicate that increased expression of TLR4 on monocytes from PE patients results in an exaggerated response to TLR4 ligands.

## Discussion

Evidence from PE patients indicates that their circulating monocytes display an aberrant activation status, with altered expression levels of surface antigens, cytokines and reactive oxygen species when compared with healthy pregnancy controls [11]–[14]. This report supports previously published findings and provides additional data describing the proportional and phenotypic differences between monocyte subpopulations of PE and NP women. We found that the non-classical, CD14^low^CD16^+^ monocyte subpopulation is increased in PE patients where they exhibit lower expression levels of HLA-DR and TIE2, and higher levels of CCR5 and TLR4.

The elevation of CD14^low^CD16^+^ monocytes has been reported in other diseases such as rheumatoid arthritis, atherosclerosis, Kawasaki disease, septic shock and human immunodeficiency virus (HIV) infection [27]–[33]. As such, expansion of non-classical monocytes may be a general phenomenon of inflammatory and infectious diseases. However, their role in the manifestation or propagation of these diseases is unclear, as very little is known about CD14^low^CD16^+^ monocyte function. After adoptive transfer into immunocompromised mice, these cells attach to the endothelium, crawl along vessels and exhibit a ‘patrolling’ behavior whereas classical and intermediate monocytes do not [23]. CD14^low^CD16^+^ monocytes from healthy non-pregnant patients are weakly phagocytic of latex beads and give little response to LPS stimulation [23].

Our data indicate that CD14^low^CD16^+^ monocytes of PE women upregulate TLR4 and exhibit an exaggerated response to LPS. Increased TLR4 levels have also been observed on neutrophils of PE women [34], suggesting that induction of TLR4 expression on circulating myeloid cells is a specific consequence of PE pathophysiology. It is tempting to speculate that a trigger factor(s) released by an abnormally ischemic placenta may be inducing early monocyte maturation – such as CD14^high^CD16^–^ monocyte differentiation into CD14^low^CD16^+^ monocytes – and/or upregulation of TLR4. In support of this notion, placental microparticles, namely syncytiotrophoblast basement membrane fragments derived from human term placentas, activate cytokine release from peripheral blood monocytes [35] and plasma from PE patients can induce ICAM-1, an adhesion receptor, in a monocytic cell line [36]. It will be interesting to determine whether TLR4 expression on newly recruited decidual macrophages correlates with TLR4 expression on circulating monocytes and whether signalling through this receptor exacerbates PE in murine models.

In contrast to our findings and others [34], studies have also reported reduced expression of *TLR4* mRNA in neutrophils of PE women [37] and impaired secretion of TNFα after LPS and PDG treatment of PE monocytes [38]. The results from these studies may be difficult to compare as gestational age, severity of the disease (i.e. proteinuria), gravidity, and genetic background of each patient population is dramatically different. Moreover, the concentration of TLR4 ligands as well as the bacterial source of the ligands is widely different among various studies. These dissimilarities strongly suggest caution is warranted when interpreting this type of data.

In addition to TLR4, we observed differences in CCR5, HLA-DR, and TIE2 between PE and NP patients; although, the ramifications of their altered expression is also unclear. CCR5 binds the chemokine CCL5/RANTES which activates signalling pathways leading to migration. In our study, CCR5 upregulation was specific to the intermediate CD14^high^CD16^+^ monocytes, indicating that this subpopulation may be recruited by CCL5 expression in the placenta. HLA-DR was found at reduced levels in PE and may affect T cell activation in these patients, as this molecule is important for antigen presentation. Of note, TIE2-expressing monocytes have been shown to promote angiogenesis in mouse tumor models [25], [39], so their decrease in PE could have major implications for the reduced oxygenation observed in PE. All of these hypotheses remain to be tested.

In conclusion, the alterations of monocyte subpopulations seen in PE with predominance of CD14^low^CD16^+^ monocytes may result in the disturbance of decidual leukocyte distribution and function. Also, the upregulation of TLR4 suggests a role for this pattern recognition receptor in the exaggerated systemic inflammatory response seen in PE but it is unclear whether this occurs as a cause or consequence of PE.
